# Tripchlorolide May Improve Spatial Cognition Dysfunction and Synaptic Plasticity after Chronic Cerebral Hypoperfusion

**DOI:** 10.1155/2019/2158285

**Published:** 2019-02-24

**Authors:** Zhao-Hui Yao, Xiao-li Yao, Shao-feng Zhang, Ji-chang Hu, Yong Zhang

**Affiliations:** ^1^Department of Geriatrics, Renmin Hospital of Wuhan University, #238 Jiefang Road, Wuhan, China; ^2^Department of Neurology, Central Hospital of Zhengzhou, #195 Tongbo Road, Zhengzhou, China; ^3^Department of Neurology, Renmin Hospital of Wuhan University, #238 Jiefang Road, Wuhan, China; ^4^Department of Pathology, Renmin Hospital of Wuhan University, #238 Jiefang Road, Wuhan, China

## Abstract

Chronic cerebral hypoperfusion (CCH) is a common pathophysiological mechanism that underlies cognitive decline and degenerative processes in dementia and other neurodegenerative diseases. Low cerebral blood flow (CBF) during CCH leads to disturbances in the homeostasis of hemodynamics and energy metabolism, which in turn results in oxidative stress, astroglia overactivation, and synaptic protein downregulation. These events contribute to synaptic plasticity and cognitive dysfunction after CCH. Tripchlorolide (TRC) is an herbal compound with potent neuroprotective effects. The potential of TRC to improve CCH-induced cognitive impairment has not yet been determined. In the current study, we employed behavioral techniques, electrophysiology, Western blotting, immunofluorescence, and Golgi staining to investigate the effect of TRC on spatial learning and memory impairment and on synaptic plasticity changes in rats after CCH. Our findings showed that TRC could rescue CCH-induced spatial learning and memory dysfunction and improve long-term potentiation (LTP) disorders. We also found that TRC could prevent CCH-induced reductions in N-methyl-D-aspartic acid receptor 2B, synapsin I, and postsynaptic density protein 95 levels. Moreover, TRC upregulated cAMP-response element binding protein, which is an important transcription factor for synaptic proteins. TRC also prevented the reduction in dendritic spine density that is caused by CCH. However, sham rats treated with TRC did not show any improvement in cognition. Because CCH causes disturbances in brain energy homeostasis, TRC therapy may resolve this instability by correcting a variety of cognitive-related signaling pathways. However, for the normal brain, TRC treatment led to neither disturbance nor improvement in neural plasticity. Additionally, this treatment neither impaired nor further improved cognition. In conclusion, we found that TRC can improve spatial learning and memory, enhance synaptic plasticity, upregulate the expression of some synaptic proteins, and increase the density of dendritic spines. Our findings suggest that TRC may be beneficial in the treatment of cognitive impairment induced by CCH.

## 1. Introduction

Chronic cerebral hypoperfusion (CCH) is a critical mechanism in the development of vascular cognitive impairment and dementia. It is the common underlying pathophysiological mechanism that contributes to cognitive decline and degenerative processes in dementia and other neurodegenerative diseases [1]. CCH promotes the progression of vascular cognitive impairment to dementia and accelerates the development of Alzheimer's disease (AD). Low cerebral blood flow (CBF) in CCH changes the homeostasis of hemodynamics and reduces the availability of oxygen, glucose, and other nutrients in the brain. This leads to disturbances in the homeostasis of energy metabolism [2, 3], which in turn leads to cerebrovascular remodeling, degeneration of the neurovascular unit and trophic coupling [4], energy loss in neurons, and vulnerability to the internal and external environment.

Previous studies have shown that CCH exacerbates neurodegeneration via multiple mechanisms, including the induction of oxidative stress which involves fatty acids, proteins, DNA, and mitochondria, blood-brain barrier disruption, increases in neuronal Ca^2+^ [5], A*β* accumulation and aggravation [6], tau hyperphosphorylation, synaptic dysfunction, neuronal loss, white matter lesions, release of neuroinflammatory cytokines [7–9], excessive autophagy [10], and overactivation of microglia in the hippocampus [11, 12]. These events lead to mitochondrial dysfunction via activation of mitophagy, changes in mitochondrial morphology due to imbalance in fusion and fission events [10, 13, 14], disturbances in lipid metabolism [15], disruption of the integrity of the white matter and fiber disarrangement of the white matter [16, 17], alterations in growth factor expression [15], inhibition of neurogenesis [18], and neurotransmitter system dysfunction [2]. Furthermore, CCH can lead to the downregulation of synaptic proteins and demyelination and the reduction of dendritic spines in the hippocampus, which then leads to a reduction in synaptic transmission and neuroplasticity [12, 19, 20]. Eventually, these pathophysiological mechanisms can result in the development of cognitive dysfunction.

Tripchlorolide (TRC), an herbal extract of Tripterygium, is a small molecule that is modified by chloride and has a molecular weight of 397. It has potent anti-inflammatory and immunosuppressive functions. Given that TRC has good lipophilicity and small molecular weight characteristics, TRC can easily pass through the blood-brain barrier and play a role in the brain [21–23]. Treatment with TRC may inhibit lipopolysaccharide-induced release of inflammatory proteins in the brain [24]. TRC may also suppress BACE1 activity which may attenuate *β*-amyloid generation [25], as well as protect neurons from microglia-mediated beta-amyloid neurotoxicity by attenuating neuroinflammatory responses [23]. Moreover, TRC has been shown to protect dopaminergic neurons from neurotoxicity induced by 1-methyl-4-phenyl-1,2,3,6-tetrahydropyridine (MPTP) in a Parkinson's disease (PD) model and prevent the reduction in dopamine levels in the striatum [26]. This neuroprotective effect is thought to be due to the anti-inflammatory and antioxidant properties of TRC [22]. In addition, TRC treatment also ameliorated defective spatial learning and memory and increased the expression of synapse-related proteins in familial AD (5XFAD) mice [27]. TRC has also been shown to improve age-associated cognitive deficits, impaired hippocampal long-term potentiation (LTP), and synapse-related receptor dysfunction in senescence-accelerated transgenic mice [21].

Given these neuroprotective effects, it is reasonable to hypothesize that TRC may improve CCH-induced cognitive impairment, although to date, no relevant investigations have been conducted. Therefore, in order to determine whether TRC can improve cognitive impairment induced by CCH, we examined the effect of TRC on spatial learning and memory impairment, as well as on changes in synaptic plasticity in rats exposed to CCH.

## 2. Materials and Methods

### 2.1. Antibodies and Chemicals

The mouse monoclonal antibody (mAb) against total *β*-actin used in this study was purchased from Abcam (Cambridge, CB, UK), while the rabbit polyclonal antibody (pAb) against vesicular glutamate transporter (vGLUT) used was purchased from Synaptic Systems (Göttingen, Germany). The rabbit pAb for N-methyl-D-aspartic acid (NMDA) receptor 2A (NR2A) used in this study was also obtained from Abcam (Cambridge, CB, UK). The rabbit pAb for postsynaptic density protein 95 (PSD95), postsynaptic density protein 93 (PSD93), NMDA receptor 2A (NR2A), NMDA receptor 2B (NR2B), glutamate receptor 1, and synapsin I, as well as the mouse mAb against NMDA receptor 1 (NR1), the mouse mAb against cAMP-response element binding protein (CREB), the phosphorylated CREB (p-CREB), the goat anti-rabbit IgG (H+L) secondary antibody Alexa Fluor 488 conjugate, and the goat anti-mouse IgG (H+L) secondary antibody Alexa Fluor 647 conjugate were all purchased from Cell Signaling Technology, Inc. (Beverly, MA, USA). The mouse mAb against glutamate receptor 2 (GluR2) used in the study was purchased from Millipore Corp. (Billerica, MA, USA). The goat anti-rabbit and anti-mouse IgG conjugated to IRDye™ (800CW) were purchased from LI-COR Biosciences (Lincoln, NE, USA). The BCA protein assay kit used in the study was from Pierce Chemical Company (Rockford, IL, USA).

Tripchlorolide was purchased from Seebio Biotechnology, Ltd. (Shanghai, China) and dissolved in saline to a concentration of 0.1 *μ*g/ml for use.

### 2.2. Animals, Chronic Cerebral Hypoperfusion (CCH) Model, and Drug Treatment

Adult Sprague-Dawley rats (male, 220-240 g) were obtained from Hunan SJA Laboratory Animal Co., Ltd. and were housed with accessible food and water *ad libitum*. Rats were kept on a 12 h light/dark cycle with the light on from 7:00 am to 7:00 pm. The Ethics Committee of Renmin Hospital of Wuhan University approved all animal care protocols and experiments.

For anesthesia, rats were intraperitoneally injected with chloral hydrate (0.4 g/kg). The permanent bilateral common carotid artery occlusion or two-vessel occlusion (2VO) procedure was performed as previously described [28]. After a ventral midline incision, both common carotid arteries were gently separated from the carotid sheath and vagus nerve [11] on a 37°C heating pad. The bilateral common carotid arteries were doubly ligated with a 4-0 silk suture just below the carotid bifurcation. In control rats, a similar surgery was performed but the vessel was not ligated. After the surgery was completed, the rats were kept in a room maintained at a temperature of 37°C until they recovered. A laser Doppler system was used to detect the level of blood flow and ensure that it had been reduced to 70% below normal, which is an important criterion of the 2VO model. CBF was measured in the cortices to reflect the perfusion of the whole brain before and after the 2VO surgery. After the rats were anesthetized with urethane (1.6 g/kg, i.p.), the skull skin was cut to expose the skull. The laser Doppler flowmetry probe was placed directly on the skull, 3.0 mm posterior to the bregma and 3.2 mm lateral to the midline, to record the CBF perfusion level.

On the third day following the 2VO surgery, the rats were intraperitoneally injected with 1 *μ*g TRC/kg every day for 28 days [21, 26, 27]. The same volume of saline was used for the sham-treated rats.

### 2.3. Morris Water Maze

After 30 days of cerebral hypoperfusion, all rats completed spatial memory training in the Morris water maze. The experiment was conducted as previously described [29]. The rats were trained in the water maze to find a hidden platform. This training is comprised of four trials per day with a 30 s intertrial interval between 2:00 and 8:00 pm for seven consecutive days. Each trial started with the rat placed in the middle of the outer edge of one quadrant and facing the wall of the pool and ended when the animal climbed onto the platform. Rats that could not find the platform in 60 s were guided to the platform. The Morris water maze video tracking analysis system (Shanghai, China) was used to record the activity trajectory of the rats. The swimming paths of the rats and latencies of the rats to find the hidden platform were recorded [30]. The time the rat spent before arriving at the platform during the first trial on each day over a seven-day period was recorded as the latency time. Upon removal of the platform, which occurred during the fourth trial on each day over the seven-day period, the number of times the rats passed through the platform area was recorded. The latency time and the number of times the rat crossed the platform area were used to evaluate learning ability. After one day of rest, the short-term memory retention test was performed. The platform was either present or absent, and rats were put into the third quadrant of the maze. The latency to reach the platform area, the number of times that the platform area was crossed, and the total time that the rat spent in the platform quadrant were recorded.

### 2.4. Novel Object Recognition Test (NOR Test)

The NOR test was performed as previously described [31]. The NOR test, which is based on the natural tendency to explore a novel object more than a familiar one [32], was used to evaluate short-term memory deficits. The rats were placed in a 55 cm × 55 cm × 38 cm open-field box made of black Plexiglas. On day 1, two objects were symmetrically placed in the box, and the rats were allowed to habituate to these objects. They were also allowed to explore and familiarize themselves with the open-field arena for 20 min. On day 2, two novel objects were placed at diagonal corners in the box, and the rats were allowed to explore these two similar objects for 5 min. On day 3, one of the two familiar objects from day 2 was replaced with a novel object to form a pair of novel and old objects. To evaluate the memory retention of the familiar and novel objects, the rats were allowed to explore the two objects for 5 min. The new object recognition experimental video analysis system (Shanghai, China) was used to record the time spent exploring each object. The ratio of the time spent exploring the novel or old object to the total time spent exploring both objects was calculated. The exploration discrimination index was calculated as the time exploring the novel object versus the old object over the total time spent exploring both objects ((time exploring the novel object - time exploring the old object)/(time exploring the novel object + time exploring the old object) ^∗^ 100%) [33, 34].

### 2.5. Electrophysiology

Synaptic plasticity is the critical physiological basis of learning and memory. Enhancement or weakening of synaptic plasticity can improve or reduce learning and memory abilities. LTP is an important form of synaptic plasticity. Therefore, to investigate the underlying mechanisms of cognitive impairment, we recorded the field potential of the brain in order to analyze the changes in LTP. After the spatial memory retention test, rats were anesthetized with urethane (1.6 g/kg, i.p.). The electrophysiological procedure was performed as previously described [28]. Electrodes were implanted at the following coordinates: 3.3 mm posterior to the bregma and 3.6 mm lateral to the midline for the recording electrode and 6.9 mm posterior to the bregma and 4.0 mm lateral to the midline for the stimulating electrode. The ground electrode was connected to the muscle contralateral to the electrode sites. Recordings of field excitatory postsynaptic potentials (fEPSPs) were made from pyramidal neurons of the Cornu Ammonis (CA) 3 region in response to the stimulation of the perforant path (PP). The data acquisition system was triggered simultaneously to record all events. The sampling frequency was 3 kHz for fEPSP recordings. The high frequency stimulation (HFS) protocol for inducing long-term potentiation (LTP) consisted of 10 trains of 15 stimuli (200 Hz, 0.5 mA) with 5 s intervals. This rather weak LTP induction protocol was chosen to prevent saturation of LTP and to thus allow for the possibility of detecting improvements or impairments. The slope of a 10 min fEPSP recording prior to the application of the HFS was used as the baseline fEPSP slope. LTP was measured as normalization of the 40 min fEPSP slope recorded after the application of the HFS over baseline fEPSP slope. And the relative values of post-HFS were further analyzed. Data were analyzed with Igor Pro 6.1 (WaveMetrics, Lake Oswego, Oregon) software.

### 2.6. Western Blotting

For Western blotting, rats were decapitated and the hippocampi were rapidly removed and homogenized. The extract was mixed with sample buffer, heated for 10 min and then centrifuged at 12,000 × g for 10 min at 25°C. The protein concentration was estimated using a bicinchoninic acid (BCA) kit. Proteins were separated using 10% sodium dodecyl sulfate polyacrylamide gel electrophoresis (SDS-PAGE) and were subsequently transferred onto nitrocellulose membranes. The membranes were blocked with 5% nonfat milk and probed overnight with a primary antibody at 4°C, incubated with anti-rabbit or anti-mouse IgG conjugated to IRDye™ (800CW) (1 : 10000) for 1 h at 4°C, and visualized using the Odyssey Infrared Imaging System (LI-COR Biosciences, Lincoln, NE, USA).

### 2.7. Immunofluorescence

Rats were anesthetized with an overdose of chloral hydrate (1 g/kg) and perfused. Their brains were then fixed and embedded with paraffin. Brains were cut into 5 *μ*m thick sections and placed on slides. The sections of rat brains were blocked with 0.3% H_2_O_2_ in absolute methanol for 10 min, followed by antigen retrieval with citric acid buffer. Nonspecific sites were blocked with bovine serum albumin (BSA) for 30 min at room temperature. The sections were then incubated overnight at 4°C with primary antibodies (1 : 200). After washing with PBS, the sections were subsequently incubated with Alexa Fluor 488 conjugated secondary antibody to allow for observation of neurons under a fluorescence microscope.

### 2.8. Golgi Staining

For Golgi staining, rats were anesthetized and perfused through the aorta for 30 min continuously with 100 ml 0.9% NaCl containing 0.5% NaN_2_O, 250 ml 4% paraformaldehyde solution, followed by 250 ml 4% paraformaldehyde solution containing 5% potassium bichromate and 5% chloral hydrate (Golgi stain). Brains were removed, cut into small pieces of tissue with volumes of 5 mm^3^, and postfixed in Golgi stain for three days. After rinsing three times, the dye solution on the surface of the tissue block was rinsed off and the blocks were dried using a filter paper. Subsequently, the blocks were immersed into a 1.5% silver nitrate solution for another three days. The silver nitrate solution was changed every 24 hours. Afterwards, the silver particles on the surface of the tissue block were brushed off, and the blocks were cut into 50 *μ*m thick sections. After the sections were dehydrated, cleared, and mounted, they were observed under oil immersion microscopy. Two to three dendrites from each of the 10 different cells per animal were analyzed. Spines were counted while manually changing the focus in order to identify all spines on a particular dendrite. Spine density was defined as the density of all spines counted per animal divided by the total length of the dendrite. It was expressed as the number of spines identified per *μ*m dendrite. The number of mushroom and filopodia spines was counted and divided by the total length of the dendrite and expressed as the number of spines identified per 100 *μ*m of dendrite. This was used as a measure of the density of mushroom and filopodia spines.

### 2.9. Statistics Analysis

Data were expressed as mean ± standard error of mean (SEM) and analyzed using SPSS 12.0 statistical software (SPSS Inc., Chicago, Illinois, USA). A repeated measures analysis of variance (ANOVA) was used to determine the statistical significance of differences in learning latency among the three groups. A one-way ANOVA followed by Dunnett's *t*-test was used to determine the statistical significance of differences in all other experiments. A *p* value of <0.05 was considered statistically significant.

## 3. Results

### 3.1. TRC Treatment Rescues Spatial Learning and Memory Impairment in a Morris Water Maze Test after CCH

**Figure 1:**
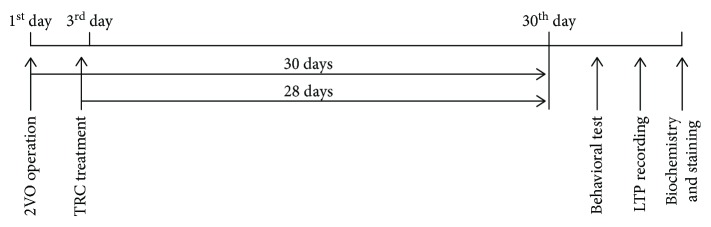
A schematic diagram of the experimental design timeline. The bilateral common carotid arteries of rats were doubly ligated just below the carotid bifurcation, which resulted in cerebral hypoperfusion. On the third day, the rats were injected intraperitoneally with 1 *μ*g tripchlorolide/kg every day for 28 days. Spatial learning and memory were then tested using the Morris water maze and novel object recognition tests. Electrophysiological tests were then performed to record the fEPSP and evaluate LTP. Finally, biochemistry, immunofluorescence, and Golgi staining were performed to detect the expression of synaptic proteins and their distribution and to evaluate the dendritic spine density.

**Figure 2:**
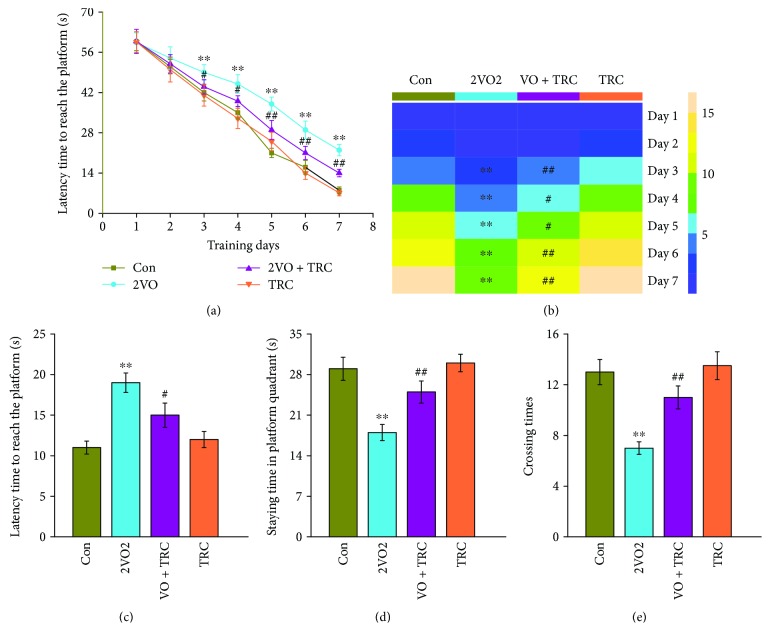
TRC treatment rescues CCH-induced spatial learning and memory impairment in the Morris water maze test. After a 30-day cerebral hypoperfusion, the rats were trained to learn and remember the location of the platform in the Morris water maze. The latency time to find the platform (a) and the number of times the platform area was crossed (b) from the first to the seventh day were recorded to evaluate the learning ability of rats. After one day of rest, the rats were retested and the latency time to find the platform (c), the time spent in the platform quadrant (d), and the number of times the platform area was crossed were recorded to evaluate short-term memory (d). Con: sham group (*n* = 12); 2VO: the group with bilateral common carotid artery ligation (*n* = 10); 2VO+TRC: the 2VO group treated with 1 *μ*g tripchlorolide/kg (*n* = 12); TRC: the sham group treated with 1 *μ*g tripchlorolide/kg (*n* = 11). Data are expressed as mean ± SEM. ^∗^*p* < 0.05, ^∗∗^*p* < 0.01 compared with the Con group, ^#^*p* < 0.05, ^##^*p* < 0.01 compared with the 2VO group.

To determine whether TRC can ameliorate cognitive impairment caused by CCH, rats were exposed to 30 days of chronic cerebral hypoperfusion induced by a 2VO surgery, followed by 28 days of TRC treatment (Figure 1), after which their spatial learning and memory abilities were examined using a Morris water maze test. The results showed that rats which underwent 2VO surgery had a noticeably longer latency time to reach the platform from the third to the seventh training day than rats which underwent sham surgery (*p* < 0.01) (Figure 2(a)). Rats which underwent 2VO surgery and TRC treatment (2VO+TRC group) had a shorter latency time from the third to the seventh training day than rats that only underwent 2VO surgery (2VO group) (days 3-4, *p* < 0.05; days 5-7, *p* < 0.01) (Figure 2(a)). During the seven-day maze learning task, the training heatmap showed that the number of times the 2VO rats crossed the platform area in 60 s was significantly less than that of the sham animals (*p* < 0.01) (Figure 2(b)), but the rats that underwent 2VO surgery and TRC treatment crossed the platform area more times than rats that underwent 2VO only (days 3, 6, and 7, *p* < 0.01; days 4-5, *p* < 0.05) (Figure 2(b)). After seven days of training and one day of rest, the short-term memory test revealed that rats which underwent 2VO surgery had a significantly longer latency time to reach the platform than sham rats (*p* < 0.01), whereas rats which underwent 2VO surgery and TRC treatment took significantly less time to reach the platform (*p* < 0.05) (Figure 2(c)). Once the platform was removed, rats which underwent 2VO surgery spent less time in the platform quadrant than sham rats (*p* < 0.01), and rats which underwent 2VO surgery and TRC treatment spent more time in the platform quadrant than rats that underwent only 2VO surgery (*p* < 0.01) (Figure 2(d)). Furthermore, rats that underwent 2VO surgery crossed the platform area fewer times than sham rats (*p* < 0.01), and rats that underwent 2VO surgery and were subsequently treated with TRC crossed the area more times than 2VO rats that did not undergo TRC treatment (*p* < 0.01) (Figure 2(e)).

### 3.2. TRC Treatment after CCH Rescues Spatial Learning and Memory Impairment in the Novel Object Recognition Test

**Figure 3:**
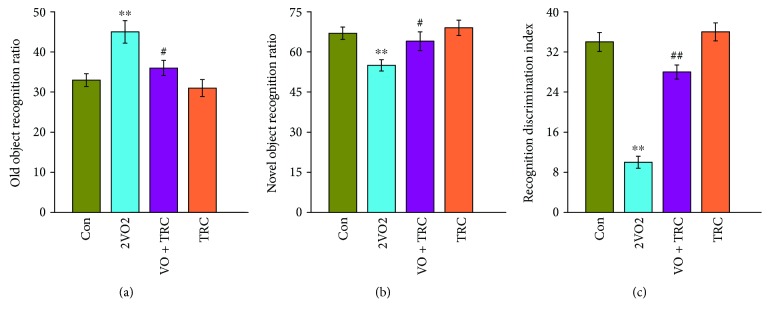
TRC treatment rescues spatial recognition learning and memory impairment in the novel object recognition test after CCH. Upon completing the Morris water maze test, the novel object recognition test was used to evaluate spatial recognition learning and memory. The time spent exploring familiar and novel objects was recorded. The ratio of the time spent exploring the novel or old object to the total time spent exploring both objects was calculated (a, b). The exploration discrimination index was calculated as the time spent exploring the novel object versus the old object to the total time spent exploring both objects (c). Con: sham group (*n* = 12); 2VO: the group with bilateral common carotid artery ligation (*n* = 10); 2VO+TRC: the 2VO group treated with 1 *μ*g tripchlorolide/kg (*n* = 12); TRC: the sham group treated with 1 *μ*g tripchlorolide/kg (*n* = 11). Data are expressed as means ± SEM. ^∗^*p* < 0.05, ^∗∗^*p* < 0.01 compared with the Con group, ^#^*p* < 0.05, ^##^*p* < 0.01 compared with the 2VO group.

In order to further understand the effect of TRC on the spatial learning and memory abilities of rats, the novel object recognition (NOR) test was used. The results showed that rats that underwent 2VO surgery spent significantly more time with the old object than the sham-treated rats (*p* < 0.01), but rats that underwent 2VO surgery and TRC treatment spent significantly less time with the old object than rats that underwent 2VO surgery only (*p* < 0.05) (Figure 3(a)). Accordingly, the rats that underwent 2VO surgery spent much less time with the new object than the sham rats (*p* < 0.01), but rats that underwent 2VO surgery and TRC treatment spent less time with the new object than rats that underwent 2VO only (*p* < 0.05) (Figure 3(b)). The final calculations revealed that rats that underwent 2VO surgery had a markedly low recognition discrimination index when compared to sham-treated rats (*p* < 0.01), but rats that underwent 2VO surgery and TRC treatment had a significantly higher recognition discrimination index than rats that underwent 2VO surgery only (*p* < 0.01) (Figure 3(c)).

### 3.3. TRC Treatment Improves the LTP Deficit after CCH

**Figure 4:**
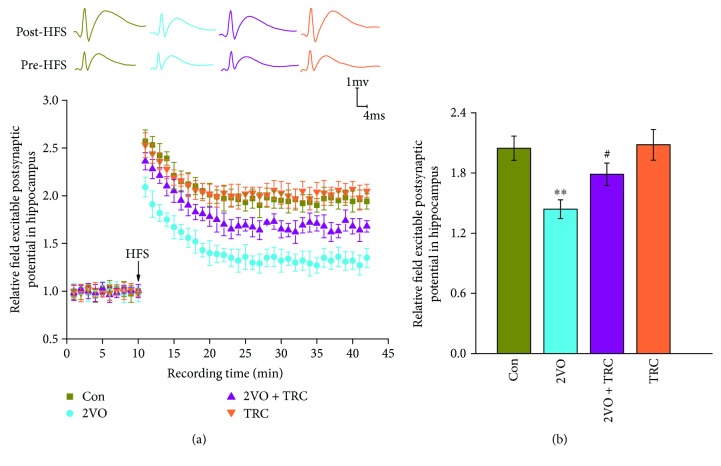
TRC treatment improves the CCH-induced LTP deficit. After completing the behavioral tests, the rats were anesthetized, and recording and stimulating electrodes were implanted with stereotaxic localization. The baseline fEPSP and the fEPSP after HFS were recorded (a). The relative fEPSP slope was then calculated (b). Con: sham group (*n* = 11); 2VO: the group with bilateral common carotid artery ligation (*n* = 10); 2VO+TRC: the 2VO group treated with 1 *μ*g tripchlorolide/kg (*n* = 10); TRC: the sham group treated with 1 *μ*g tripchlorolide/kg (*n* = 11). Data are expressed as means ± SEM. ^∗^*p* < 0.05, ^∗∗^*p* < 0.01 compared with the Con group, ^#^*p* < 0.05, ^##^*p* < 0.01 compared with the 2VO group.

Long-term potentiation (LTP) reflects synaptic plasticity which was the physiological basis for hippocampus-dependent spatial learning and memory [35] and is indispensable for hippocampus-dependent spatial learning and the formation and retrieval of hippocampus-dependent spatial memory [36]. To investigate the underlying electrophysiological mechanisms of the effect of TRC on cognitive impairment induced by CCH, we performed *in vivo* electrophysiology. After high frequency stimulation (HFS), we recorded the field excitatory postsynaptic potentials (fEPSPs). The fEPSP amplitudes were remarkably lower in the 2VO group ((145.38 ± 9.29) %) than in the sham group ((206.72 ± 12.45) %) (*p* < 0.01), but the fEPSP amplitudes were significantly higher in the 2VO+TRC group ((180.75 ± 11.08) %) than in the 2VO group (*p* < 0.05) (Figures 4(a) and 4(b)).

### 3.4. TRC Treatment Prevents CCH-Induced Downregulation of Synaptic Proteins

**Figure 5:**
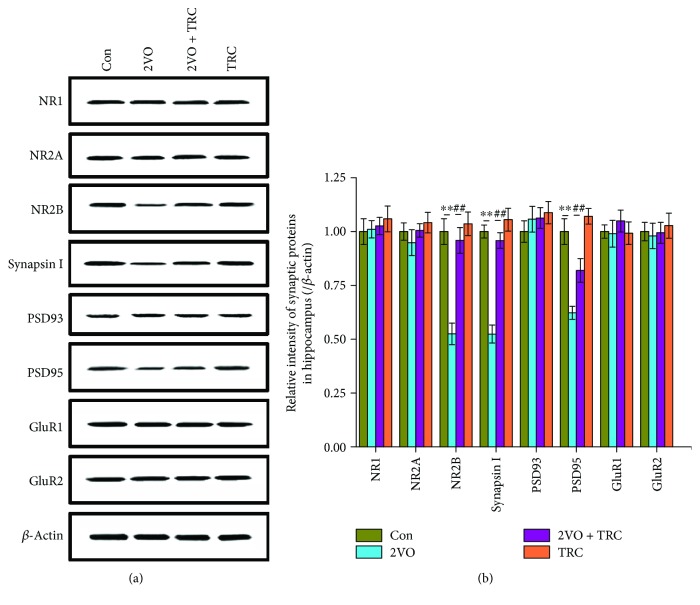
TRC treatment prevents the CCH-induced downregulation of synaptic proteins. After the electrophysiological tests, some rats were euthanized and their brain tissues were homogenized for Western blot analysis of NR1, NR2A, NR2B, synapsin I, PSD93, PSD 95, GluR1, GluR2, and *β*-actin (a), and the relative intensity of protein bands was normalized to *β*-actin (b). Con: sham group (*n* = 3); 2VO: the group with bilateral common carotid artery ligation (*n* = 4); 2VO+TRC: the 2VO group treated with 1 *μ*g tripchlorolide/kg (*n* = 4); TRC: the sham group treated with 1 *μ*g tripchlorolide/kg (*n* = 3). Data are expressed as means ± SEM. ^∗^*p* < 0.05, ^∗∗^*p* < 0.01 compared with the Con group, ^#^*p* < 0.05, ^##^*p* < 0.01 compared with the 2VO group.

Synaptic proteins are important components of synaptic structure formation. They are involved in vesicle synthesis, transport, and release and normal synaptic function. Synaptic protein expression is critical for synaptic plasticity and the maintenance of normal learning and memory [37]. To explore the potential molecular mechanism of CCH-induced learning and memory impairment, we used Western blotting to examine the expression of synapse-related molecules. Our data showed that NR1, NR2A, PSD93, GluR1, and GluR2 in the 2VO group did not change significantly compared to those in the sham group (*p* > 0.05) (Figures 5(a) and 5(b)). The NR2B, synapsin I, and PSD95 levels in the 2VO group were significantly lower than those in the sham group (*p* < 0.01), but the expression of these same proteins in the 2VO+TRC group was significantly higher than in the 2VO group (*p* < 0.01) (Figures 5(a) and 5(b)).

### 3.5. TRC Treatment Prevents CCH-Induced Downregulation of Phosphorylated CREB

**Figure 6:**
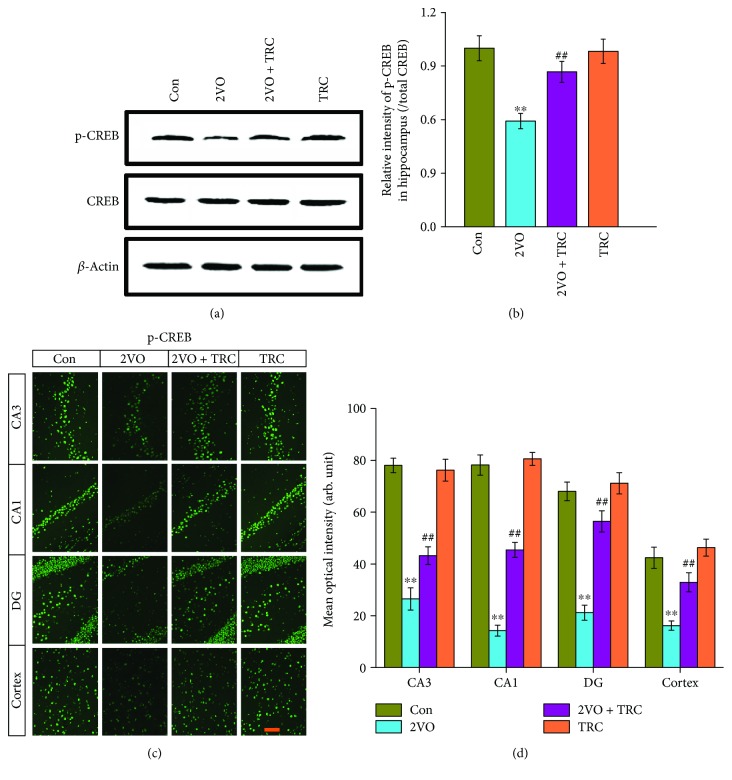
TRC treatment prevents the CCH-induced downregulation of phosphorylated CREB. CREB expression was detected by Western blotting (a), and the relative intensity of the band was normalized to *β*-actin (b). Brains were also fixed with 4% paraformaldehyde, embedded with paraffin, cut into 5 *μ*m thick sections, and placed on slides. The sections were incubated with the CREB and p-CREB antibody to detect CREB and p-CREB distribution (c), and the mean optical intensity of p-CREB positive neurons in the CA1 and CA3 regions of the hippocampus, dentate gyrus (DG), and cortex was measured and analyzed (d). Bar = 50 *μ*m. Con: sham group (*n* = 3); 2VO: the group with bilateral common carotid artery ligation (*n* = 4); 2VO+TRC: the 2VO group treated with 1 *μ*g tripchlorolide/kg (*n* = 4); TRC: the sham group treated with 1 *μ*g tripchlorolide/kg (*n* = 3). Phosphorylated CREB: p-CREB. Data are expressed as means ± SEM. ^∗^*p* < 0.05, ^∗∗^*p* < 0.01 compared with the Con group, ^#^*p* < 0.05, ^##^*p* < 0.01 compared with the 2VO group.

CREB is a transcription factor that controls the expression of many synaptic proteins. It plays a key role in neuronal excitability and controls the hippocampus and cortex plasticity circuits [38, 39]. CREB is very important for memory formation [40]. Moreover, CREB downregulation leads to cognitive decline [41, 42]. To clarify whether CREB plays a role in the spatial cognitive impairment induced by CCH, we examined CREB expression using Western blotting. We did not find any change in the CREB level. Because phosphorylated CREB (p-CREB) is the active form of CREB, we further evaluated the p-CREB level. The data showed that p-CREB levels in rats which underwent 2VO surgery were significantly lower than those in sham-treated rats (*p* < 0.01), but rats that underwent 2VO surgery and TRC treatment had much higher p-CREB levels than rats that underwent 2VO surgery only (*p* < 0.01) (Figures 6(a) and 6(b)). To further understand the distribution of p-CREB in the subregions of the hippocampus, we performed immunofluorescence staining of p-CREB in brain slices and found that rats that underwent 2VO surgery had a significantly lower mean optical intensity of p-CREB staining in the CA3 and CA1 regions, dentate gyrus, and cortex than sham-treated rats (*p* < 0.01), whereas rats that underwent 2VO surgery and TRC treatment had a significantly higher mean optical intensity of p-CREB staining in these regions (*p* < 0.01) (Figures 6(c) and 6(d)).

### 3.6. TRC Treatment Rescues the Reduction in Dendrite Spine Density after CCH

**Figure 7:**
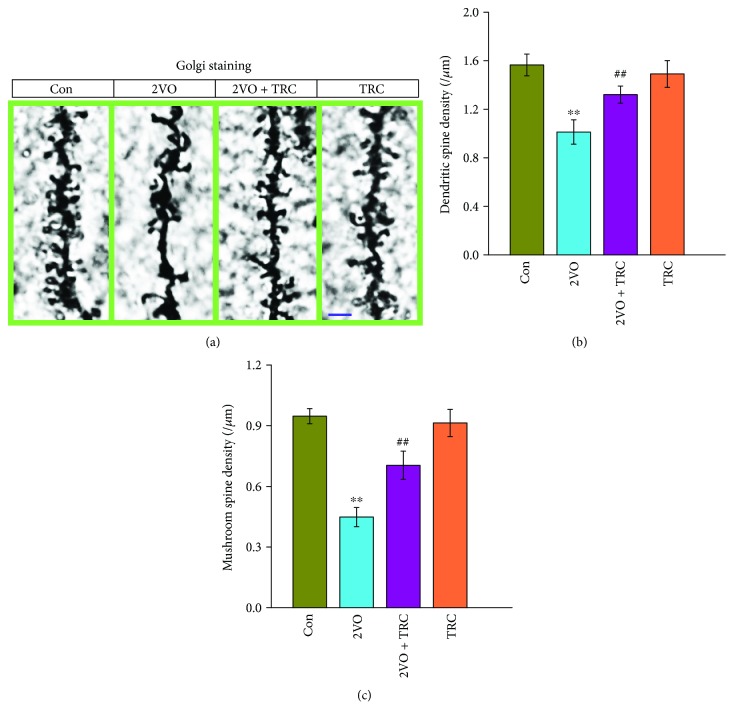
TRC treatment rescues the CCH-induced reduction in dendrite spine density. Rats were anesthetized and perfused through the aorta with a solution containing both Golgi staining and the perfusion solution for fixation. The brain tissue block was then stained with the Golgi staining solution for three days, cut into 50 *μ*m thick slices, and placed on the slide for observation of dendritic spine under the microscope (1000x) (a). The dendritic spines were then counted and the density of dendritic spines and mushroom spines were calculated (b, c). Bar = 2.5 *μ*m. Con: sham group (*n* = 3); 2VO: the group with bilateral common carotid artery ligation (*n* = 3); 2VO+TRC: the 2VO group treated with 1 *μ*g tripchlorolide/kg (*n* = 3); TRC: the sham group treated with 1 *μ*g tripchlorolide/kg (*n* = 3). Data are expressed as means ± SEM. ^∗^*p* < 0.05, ^∗∗^*p* < 0.01 compared with the Con group, ^#^*p* < 0.05, ^##^*p* < 0.01 compared with the 2VO group.

Dendritic spines are important locations for the formation of neuronal circuits and network structures. A reduction in dendritic spine density is bound to reduce synapse formation and thus impair cognitive function [43]. Our previous study showed that CCH can lead to a reduction in dendritic spines. We thus chose to investigate whether TRC treatment could regulate the density and morphology of dendritic spines after CCH by using the Golgi stain to label and display dendritic spines. The data showed that rats that underwent 2VO surgery had a noticeably lower density of dendritic spines in hippocampal neurons than sham-treated rats (*p* < 0.01), but the rats that underwent 2VO surgery and TRC treatment had a higher density of dendritic spines than rats that underwent 2VO surgery only (*p* < 0.01) (Figures 7(a) and 7(b)). Mushroom spines are mature spines and are necessary for neuronal synapse formation. The density of mushroom spines can determine the number of synapses that have formed. Our data showed that rats which underwent 2VO surgery had a noticeably lower density of mushroom spines than sham-treated rats (*p* < 0.01), whereas rats that underwent 2VO surgery and TRC treatment had a higher density of mushroom spines than rats which underwent 2VO surgery only (*p* < 0.01) (Figures 7(c) and 7(d)).

## 4. Discussion

In the present study, we show that TRC can rescue CCH-induced spatial learning and memory dysfunction and improve LTP disorders. Additionally, we found that TRC can prevent the reduction in NR2B, synapsin I, and PSD95 expression that occurs in response to CCH. Moreover, TRC leads to an upregulation in p-CREB levels. p-CREB is an important transcription factor for synaptic proteins. TRC prevented the CCH-induced reduction in dendritic spine density.

LTP of synaptic transmission is a form of long-term synaptic plasticity [44] and is important for circuit refinement during memory formation and behavioral changes [45]. LTP can be quickly induced with a persistent increase in synaptic efficacy after high frequency stimulation [46]. Learning can enhance LTP [47], and LTP have also been shown to enhance learning and memory in transgenic models [48]. Impairment of LTP can lead to spatial memory deficits [49]. Alleviating deficits in hippocampal long-term potentiation improved memory in a rat model of Alzheimer's disease [50]. In our study, we found that TRC treatment improves CCH-induced LTP impairment, which suggests that TRC increased the efficacy of synaptic transmission in neuronal circuits and rescued the CCH-induced synaptic plasticity deficits. Previous studies have shown that TRC can stimulate brain-derived neurotrophic factor (BDNF) mRNA expression [26]. Endogenously secreted BDNF affects synaptic plasticity [51], regulates synaptic transmission and long-term potentiation (LTP) in the hippocampus, and plays a role in the formation of memory [52]. This suggests that TRC may improve the CCH-induced impairment in synaptic plasticity by regulating the expression of hippocampal endogenous BDNF. This will be explored and verified in future research. In our previous study, we demonstrated that chronic neuroinflammation in brain tissues after CCH treatment can impair cognition. Moreover, CCH induces memory impairment and accelerates A*β* generation [9, 53]. Interestingly, TRC produces anti-inflammatory effects by downregulating extracellular signal-regulated protein kinases 1/2 (ERK1/2) nuclear factor kappa-light-chain-enhancer of activated B cells (NF-*κ*B) and JAK/STAT signaling pathways [24, 54]. TRC also attenuates A*β* generation by inhibiting BACE1 activity and protects neurons against microglia-mediated A*β* neurotoxicity by suppressing NF-*κ*B and JNK signaling [23, 25]. These indicate that TRC is a potentially effective neuroprotective agent. In future research, we will conduct systematic analyses on the anti-inflammatory effects of TRC in CCH.

N-Methyl-D-aspartate receptors (NMDARs) are fundamental to learning, memory, and excitatory postsynaptic potential. Triheteromeric NR1/NR2A/NR2B receptors constitute the major NMDARs in adult hippocampal synapses [55, 56]. NR1 is important for the maintenance of normal cognition and reduction in the NMDAR-subunit NR1 (also known as GluN1) that impairs spatial reference memory [56, 57]. The antibody against NR1 contributed to severe cognitive impairment [58]. In our study, we did not observe changes in NR1 after CCH, which suggests that NR1 is not involved in spatial learning and memory impairment after CCH. NR2A is required for memory, and attenuation of the expression of NR2A has been shown to be associated with cognitive decline [59]. Given that the level of NR2A did not change in our study, NR2A may also not be involved in CCH-induced cognitive impairment. The NMDAR subunit NR2B is important for synaptic plasticity and memory. An increase in the surface expression of NR2B has been shown to facilitate synaptic transmission and improve memory formation *in vivo* [60]. NR2B degradation has also been shown to impair synaptic plasticity and learning [61]. In contrast, upregulation of the expression of the NR2B subunit can enhance synaptic plasticity and memory function [62]. The current study suggests that a reduction in NR2B levels may be involved in CCH-induced spatial cognitive impairment and that TRC can upregulate the expression of NR2B. Previous studies also showed that TRC could upregulate synapse-related proteins including the NMDA receptor and improve cognition impairment in an AD transgenic model [21, 27]. This implies that TRC may improve spatial memory and synaptic plasticity by upregulating the expression of NR2B. Synapsin I is an important presynaptic protein that is located on synaptic vesicles and contributes to neurotransmitter release [63, 64], which, in turn, regulates synaptic plasticity and memory strength [65, 66]. Postsynaptic density protein 95 (PSD95) and PSD93, major postsynaptic proteins, are also critical for synaptic plasticity [67]. Deficient synapsin I, PSD95, and PSD93 have been shown to be associated with cognitive impairments [68–70]. In our study, synapsin I and PSD95, but not PSD93 levels were reduced by CCH. This reduction, however, was prevented by TRC treatment, which suggests that synapsin I and PSD95 may be involved in the CCH-induced cognitive impairment and the protective effects of TRC treatment.

GluR1 and GluR2 are major subunits of important excitable glutamate amino-acid-3-hydroxy-5-methyl-isoxazol-4-propionic acid receptors (AMPARs), which regulate synaptic plasticity and memory [71, 72]. GluR1 and GluR2 levels were not altered in our study. However, the activity of GluR2A and GluR2 is regulated by phosphorylation, and their involvement in CCH-induced cognitive impairment can therefore not be excluded. Future investigations should therefore seek to address whether GluR2A and GluR2 are involved in CCH-induced cognitive impairment.

Synaptic plasticity can mediate memory storage [73], during which new mRNA and protein syntheses are required [74]. The transcription factor CREB regulates many genes involved in synaptic plasticity [75] and is critical for hippocampus-dependent learning and memory [76]. Inhibition or downregulation of CREB can lead to cognitive impairment [73, 77]. Our studies have shown that TRC can prevent the CCH-induced inhibition of CREB, which suggests that TRC may enhance the transcription and synthesis of learning and memory-related proteins in hippocampal neurons after CCH, thereby improving synaptic plasticity and cognitive dysfunction.

Dendritic spines are postsynaptic structural components of excitatory synapses that receive excitatory input from axons at the synapse. Dendritic spines mediate transmission of electrical signals to the neuron. They contain NMDA receptors and AMPA receptors [78]. The dendritic spine geometry is AMPA receptor and NMDA receptor-dependent Ca^2+^ signaling currents in dendrites [79–81]. In our study, we observed a reduction in the expression of the NMDA receptor subunit NR2A. This suggests that the level of NR2A in the dendritic spine may be reduced, which would thus affect the excitatory signal current that is mediated by the dendritic spine. To our knowledge, however, this has not yet been investigated, and further experiments are needed for clarification. Dendritic spines are also important sites for neuronal plasticity and synaptic activity that induces hippocampal dendritic morphogenesis [82, 83]. Long-term enhancement of hippocampal synaptic efficacy promotes the formation of new spines while inhibition of long-term potentiation significantly reduces the number of new spines that are generated [84]. Hippocampal activity-dependent structural remodeling, namely, structural plasticity, can reconstruct neural circuits and has been regarded as a critical cellular basis for learning, memory, and synaptic plasticity [85]. Normal brain function is required for the regulation of the balance between spine formation and spine elimination [86]. Excessive dendritic spine elimination leads to a reduction in spines, which contributes to defective hippocampus-dependent memory [87]. On the contrary, an increase in the density of dendritic spines can improve memory and neuronal plasticity [88]. The present study shows that treatment with TRC can prevent the CCH-induced reduction in the density of dendritic spines, which suggests that TRC treatment can prevent deficits in the structural plasticity of hippocampal neurons that are caused by CCH. Structural plasticity is closely correlated with learning and memory ability and synaptic plasticity. This also suggests that TRC treatment improves structural plasticity and thus improves one of the mechanisms by which CCH leads to cognitive impairment.

## 5. Conclusion

We found that TRC can improve spatial learning, memory, and synaptic plasticity; upregulate the expression of several synaptic proteins; and increase the density of dendritic spines. Our findings suggest that administration of TRC may be an important therapy for the treatment of CCH-induced cognitive impairment.

## Data Availability

The datasets in the current study are available from the corresponding author upon reasonable request.
